# Predicted Disappearance of *Cephalantheropsis obcordata* in Luofu Mountain Due to Changes in Rainfall Patterns

**DOI:** 10.1371/journal.pone.0029718

**Published:** 2012-01-10

**Authors:** Xin-Ju Xiao, Ke-Wei Liu, Yu-Yun Zheng, Yu-Ting Zhang, Wen-Chieh Tsai, Yu-Yun Hsiao, Guo-Qiang Zhang, Li-Jun Chen, Zhong-Jian Liu

**Affiliations:** 1 Shenzhen Key Laboratory for Orchid Conservation and Utilization, The National Orchid Conservation Center of China/The Orchid Conservation & Research Center of Shenzhen, Shenzhen, China; 2 Continuing Education College of Beijing Forestry University, Beijing, China; 3 The Center for Biotechnology and BioMedicine, Graduate School at Shenzhen, Tsinghua University, Shenzhen, China; 4 Institute of Tropical Plant Sciences, and Orchid Research Center, National Cheng Kung University, Taiwan, China; 5 Department of Life Sciences, National Cheng Kung University, Taiwan, China; 6 College of Forestry, South China Agricultural University, Guangzhou, China; DOE Pacific Northwest National Laboratory, United States of America

## Abstract

**Background:**

In the past century, the global average temperature has increased by approximately 0.74°C and extreme weather events have become prevalent. Recent studies have shown that species have shifted from high-elevation areas to low ones because the rise in temperature has increased rainfall. These outcomes challenge the existing hypothesis about the responses of species to climate change.

**Methodology/Principal Findings:**

With the use of data on the biological characteristics and reproductive behavior of *Cephalantheropsis obcordata* in Luofu Mountain, Guangdong, China, trends in the population size of the species were predicted based on several factors. The response of *C. obcordata* to climate change was verified by integrating it with analytical findings on meteorological data and an artificially simulated environment of water change. The results showed that *C. obcordata* can grow only in waterlogged streams. The species can produce fruit with many seeds by insect pollination; however, very few seeds can burgeon to become seedlings, with most of those seedlings not maturing into the sexually reproductive phase, and grass plants will die after reproduction. The current population's age pyramid is kettle-shaped; it has a Deevey type I survival curve; and its net reproductive rate, intrinsic rate of increase, as well as finite rate of increase are all very low. The population used in the artificial simulation perished due to seasonal drought.

**Conclusions:**

The change in rainfall patterns caused by climate warming has altered the water environment of *C. obcordata* in Luofu Mountain, thereby restricting seed burgeoning as well as seedling growth and shortening the life span of the plant. The growth rate of the *C. obcordata* population is in descending order, and models of population trend predict that the population in Luofu Mountain will disappear in 23 years.

## Introduction

The past century has witnessed an increase of approximately 0.74°C in the global average temperature and extreme weather events. Many predictions have been made about the responses of species to climate change [1]–[8], such as their non-response or local extinction, disappearance or reduction in suitable distribution ranges, shift of expansion or distribution ranges poleward or toward higher elevations to inhabit areas within their metabolic temperature tolerances, and variations in phenology and behavior, even in genes. Recent studies have shown that species have shifted to low-elevation areas from high ones because the rise in temperature has increased rainfall [9]. As these outcomes challenge the hypothesis about the perceived responses of species to climate change, the capability of wild species to respond to climate warming, especially to the accompanying extreme weather events, should be investigated.

Orchids usually thrive in climax communities and are very sensitive to environmental changes [10]. *Cephalantheropsis obcordata* belongs to the family Orchidaceae and has special requirements for habitation. It exists in South Fujian, Guangdong, Hainan, Taiwan, and the southern and southeastern parts of Yunnan in China, as well as in Japan, India, Indonesia, Laos, Malaysia, Myanmar, the Philippines, Thailand, and Vietnam [11]. In China, it grows in moist sloping fields where stones have humus soil beside streams in climax subtropical forest communities with higher canopy density [11]. Climate changes directly influence the existence and development of *C. obcordata*, rendering the effects of global warming on the population growth and decline of this species an important area of study.

Much research into the population development trends of endangered species, the establishment of conservation strategy and measures, and the reasonable utilization of current resources by analyzing population quantities has been carried out [12]–[16], but data on how climate change specifically affects orchid plants, particularly the trends in their population development, are limited [17], [18]. With the use of data on the biological characteristics and pollination biology of *C. obcordata*, this current study predicted the dynamic population trends of the species based on its static life and reproduction tables, survival curve, and age pyramid. It also estimated changes in those trends using the Leslie matrix model and the Levins model. This report discusses the spatial pattern, age pyramid, breeding strategy, and population size trends of *C. obcordata* in light of its extinction vulnerability from global warming. The data highlight the need to protect this species while integrating climate change considerations.

## Results

### Biological characteristics of *C. obcordata*

#### Growth characteristics and space–time mark

**Figure 1:**
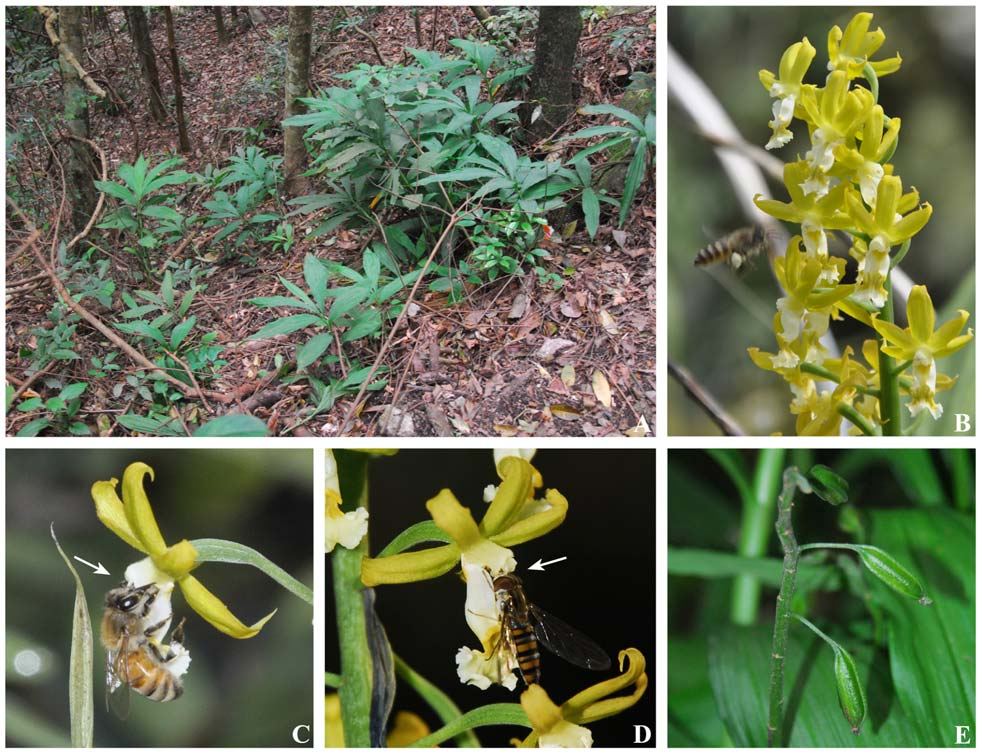
Growth condition and spatial structure of *C. obcordata*. (**A**) Porphyritic wild populations. (**B**) An inflorescence. (**C**) A bee *Apis cerana* visits the flower (arrow). (**D**) A hoverfly *Betasyrphus serarius* visits the flower (arrow). (**E**) Fruited plant.

*C. obcordata* is a proximate hygrophyte that grows on shadowly wooded slopes with deep loose humus soil often soaked with water in mountain valleys (Figure 1). Its genet has rhizomes that can grow a number of ramets. New ramets grow from the base of the youngest ramet in early May each year, and a chain-like plant forms year after year. Seedlings need 3 years of vegetative growth to blossom, which usually occurs in the fourth year, followed by sexual reproduction. Floral bud differentiation begins in August, florescence starts in late September and ends in December, and fruiting occurs between November and March. The fruit will split and scatter seeds at maturity, each seed will produce protocorm in humus soil and grow a short stem in the coming year, and a bud will grow from the stem base and then become a new ramet by the following year. Each genet grows only one new ramet annually, representing an obvious space–time mark. The flowering rate of sexually mature genets was 30.24%±9.61% in this study (*n* = 6).

#### Spatial pattern

In sample grids of *C. obcordata*, the occurrence frequencies of a square with very few individuals and that with many individuals were higher than the expected values in Poisson distribution, that is, 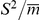 = 1.35, 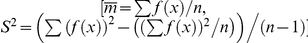, which was clearly higher than 1. The spatial structure of *C. obcordata* was thus of clumped distribution [19]. Such spatial pattern is correlated with its habitat adaptability and seed scattering. *Habitat adaptability* refers to the species' requirement for waterlogging in its habitat — such habitat exists only in the buffer section of streams and hills with water seepage, which characterize the spatial distribution pattern of *C. obcordata*; the species hence usually has concentrated growth in narrow places and mutually forms different maculated populations (Figure 1A) that are spatially separated. On the other hand, *seed scattering*, as the term itself suggests, refers to the scattering of seeds that burgeon into seedlings and observe a clumped distribution near the limited habitat of the species.

#### Age pyramid

**Figure 2:**
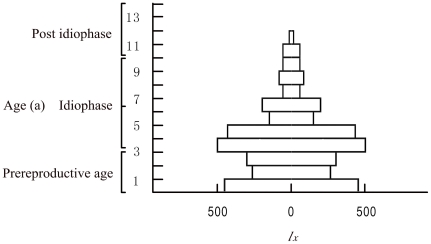
The age pyramid of the *C. obcordata* population.

Statistical data on each genet by age class revealed the following: the genets from each age class were asymmetrically distributed; the number of genets from each age class that advanced into an upper class greatly differed from the number in lower classes; the proportion of young genets was smaller, and the proportion of aged ones was the smallest, although the proportion of adult genets was very high; the death rate was higher than the birth rate; and the age pyramid was kettle-shaped, indicating that *C. obcordata* population is descending (Figure 2) [19].

#### Reproductive mechanism

Due to the clumped distribution of *C. obcordata*, the comparatively concentrated flowers had increased the efficiency of pollination (Figure 1B). Bee *Apis cerana* (Figure 1C) and hoverfly *Betasyrphus serarius* (Figure 1D) were found to visit flowers during the entire observation period; 28.32%±15.94% (*n* = 10) flowers produced fruits, with each fruit able to produce 4782±1327 (*n* = 10) seeds (Figure 1E). The fruiting rate of bagged flowers was zero, indicating that *C. obcordata* did not automatically self-pollinate and produce asexual seeds. The fruiting rates of artificial pollination and cross-pollination were both 100%, revealing that the self-pollination and cross-pollination of *C. obcordata* had affinity and that its sexual reproduction was not without obstacles.

### Population trend and quantity analysis

#### Static life table

The life table of the *C. obcordata* population was formatted based on six subpopulations and the 1-year age class (phenological cycle). Table 1 shows the static life table for this population, with the results demonstrating that the death rate of seedlings from the 1-year age class was as high as 40%, thereby indicating that more of those seedlings died when turning into 2-year age class seedlings. The death rates of seedlings from the 2- and 3-year age classes were negative, revealing that the seedlings failed to grow into the next age class and that the shortage of seedling storage was severe, as the death rate and disappearance rate (*K_x_*) of the population were rather high before the seedlings (from the 2- and 3-year age classes) reached sexual maturity. The population had trended toward degeneration because of the intense filtration of the environment. Its descending trend was consistent with its kettle-shaped age pyramid. The death rate of sexually mature plants (4-year age class) was 13.64%, and the genet rate after sexual maturity was 63.16%; the death rates of genets in subsequent age classes were very high, except for the negative rate at the 6- and 8-year age classes and the zero rate at the 10-year age class. On one hand, genets of this population had died after sexual reproduction and left the next generation with living space, indicating that individuals' demand for nutrition space had constantly increased after the reproduction phase and that mortality was very high because of the enhanced external fluffing action of the population as influenced by water and other ecological factors. On the other hand, some individuals could grow up to the oldest age class after the physiological senescence phase, occupying environmental conditions (in places with better water conditions) that could better satisfy their growth needs, and the death rate of old individuals was lower, until their physiological senescence.

**Table 1:** Static life table of the *C. obcordata* population.

Year	*X*	*a_x_*	*l_x_*	*d_x_*	*q_x_*	*L_x_*	*T_x_*	*e_x_*	ln*l_x_*	*K_x_*
2010	1	20	909.09	363.64	400.00	727.27	4863.64	5.35	6.81	0.51
2009	2	12	545.45	−90.91	−166.67	590.91	4136.36	7.58	6.30	−0.15
2008	3	14	636.36	−363.64	−571.43	818.18	3545.45	5.57	6.46	−0.45
2007	4	22	1000.00	136.36	136.36	931.82	2727.27	2.73	6.91	0.15
2006	5	19	863.64	545.45	631.58	590.91	1795.45	2.08	6.76	1.00
2005	6	7	318.18	−90.91	−285.71	363.64	1204.55	3.79	5.76	−0.25
2004	7	9	409.09	272.73	666.67	272.73	840.91	2.06	6.01	1.10
2003	8	3	136.36	−45.45	−333.33	159.09	568.18	4.17	4.92	−0.29
2002	9	4	181.82	45.45	250.00	159.09	409.09	2.25	5.20	0.29
2001	10	3	136.36	0.00	0.00	136.36	250.00	1.83	4.92	0.00
2000	11	3	136.36	90.91	666.67	90.91	113.64	0.83	4.92	1.10
1999	12	1	45.45	45.45	1000.00	22.73	22.73	0.50	3.82	3.82

#### *K*-factor

Table 1 shows that the dead genets of *C. obcordata* were mainly seedlings from the 1-year age class and that the genets reached sexual maturity. Field observations revealed that the 1-year age class seedlings and genets died immediately after fructification. In the artificial drought test, death appeared earliest in young ramets and genets after fructification. Drought possibly caused the death (*k*_alevin_) of seedlings and plants after reproduction, which is closely correlated with the total death rate (*k*_total_). The death of genets after reproduction invalidated their multireproductive ability, which directly reduced the produce of seedlings. Moreover, the change in the death rate of *C. obcordata* at the seedling phase would yield fluctuations in the total death rate and the population size.

#### Survival curve

**Figure 3:**
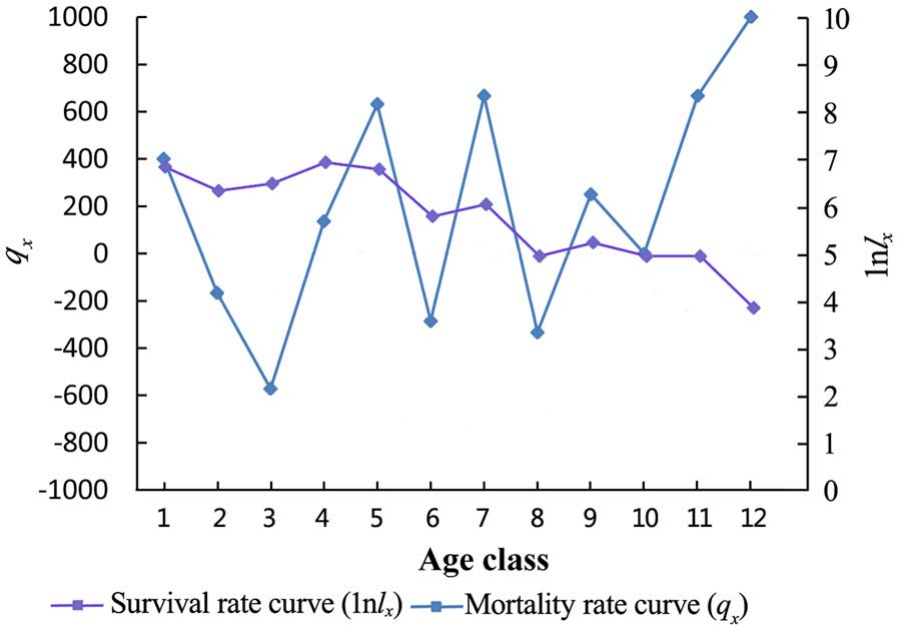
Survival rate and mortality rate curves of the *C. obcordata* population.

The survival curve and death rate curve of *C. obcordata* are illustrated in Figure 3. Figure 3 shows a Deevey type I survival curve, indicating that the survival rate of the 1-year age class seedlings was not high but that the death rate after sexual reproduction was. Figure 3 shows that the population structure was unstable and that the death rates at different age phases varied greatly. Individuals of the 1-year age class and those that were sexually mature had high death rates; however, although the surviving population had a high living quality, the individual number was very small, marking a descending trend. The survival curve and death rate curve reveal that a mass of young individuals could not enter the sexual phase successfully and that those sexually reproductive individuals could not produce a high number of young individuals during their thriving breeding period and instead die in mass, such that stability could not be sustained for the population.

#### Reproduction table and parameters

The reproduction table of *C. obcordata* is shown as Table 2. The net reproductive rate 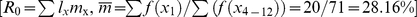 was 0.909, indicating that each generation could multiply 0.909 times. The intrinsic rate of increase (*r_m_* = ln*R_o_*/*T*) was −1.884, with *r_m_*<0 demonstrating that the instantaneous birth rate was lower than the instantaneous death rate. The finite rate of increase (*λ* = e*^r^* = e^−1.884^) was 0.152, which entails that the population had decreased geometrically at a rate of 0.152; the generation span [*T* = (Σ*Xl_x_m_x_*)/*R_o_*] was 5.966a (year), yielding the average age of 5.966a for genets in the generative phase. The result of parameters *R_0_*<1, *r_m_*<0, *λ*<1 revealed that the population of *C. obcordata* cannot completely self-renew and has been descending [20]–[22].

**Table 2:** Fecundity schedule of the *C. obcordata* population.

*X*	*l_x_*	*m_x_*	*l_x_m_x_*	*Xl_x_m_x_*
1	0.909	–	–	–
2	0.545	–	–	–
3	0.636	–	–	–
4	1.000	0.282	0.282	1.128
5	0.864	0.282	0.244	1.218
6	0.318	0.282	0.090	0.538
7	0.409	0.282	0.115	0.808
8	0.136	0.282	0.038	0.308
9	0.182	0.282	0.051	0.461
10	0.136	0.282	0.038	0.385
11	0.136	0.282	0.038	0.423
12	0.045	0.282	0.013	0.154

#### Leslie matrix model and predicted model of dynamic quantity

Each sexually reproductive genet from the six subpopulations was tested. The average number of filial seedlings produced (*m_x_* = 0.282) represented reproductive ability in calculating the Leslie matrix model and predicting the changes of population size and age structure for 20a (Table 3).

**Table 3:** Leslie matrix model of the *C. obcordata* population.

0	0	0	0.179	0.174	0.212	0.165	0.282	0.242	0.188	0.071	0.000
0.813											
	1.385										
		1.139									
			0.634								
				0.615							
					0.750						0
						0.583					
							1.000				
								0.857			
									0.667		
		0								0.250	
											0.000

*C. obcordata* is a perennial plant, and its sexual reproduction can reach the physiological time limit. However, it has weak fecundity and scarce capacity for seedling supplementation, such that the population presents a descending trend. The dynamic quantity model is given as *N_t_* = *N_t_*_–1_–*N_t_*_–1_e^−1.884^, and its predicted results are shown in Table 4.

**Table 4:** Dynamic preestimates of population size and continuous descending model of the *C. obcordata* population.

Age class	*N_0_*	*N_1_*	*N_2_*	*N_3_*	*N_4_*	*N_5_*	*N_6_*	*N_7_*	*N_8_*	*N_9_*	*N_10_*	*N_11_*	*N_12_*	*N_13_*	*N_14_*	*N_15_*	*N_16_*	*N_17_*	*N_18_*	*N_19_*	*N_20_*	*N_21_*	*N_22_*	*N_23_*
1	20	17	14	12	10	9	7	6	5	5	4	3	3	2	2	2	1	1	1	1	1	1	1	0
2	12	10	9	7	6	5	4	4	3	3	2	2	2	1	1	1	1	1	1	1	0	0	0	0
3	14	12	10	9	7	6	5	4	4	3	3	2	2	2	1	1	1	1	1	1	1	0	0	0
4	22	19	16	13	11	10	8	7	6	5	4	4	3	3	2	2	2	1	1	1	1	1	1	0
5	19	16	14	12	10	8	7	6	5	4	4	3	3	2	2	2	1	1	1	1	1	1	1	0
6	7	6	5	4	4	3	3	2	2	2	1	1	1	1	1	1	1	0	0	0	0	0	0	0
7	9	8	6	5	5	4	3	3	2	2	2	1	1	1	1	1	1	1	0	0	0	0	0	0
8	3	3	2	2	2	1	1	1	1	1	1	0	0	0	0	0	0	0	0	0	0	0	0	0
9	4	3	3	2	2	2	1	1	1	1	1	1	1	0	0	0	0	0	0	0	0	0	0	0
10	3	3	2	2	2	1	1	1	1	1	1	0	0	0	0	0	0	0	0	0	0	0	0	0
11	3	3	2	2	2	1	1	1	1	1	1	0	0	0	0	0	0	0	0	0	0	0	0	0
12	1	1	1	1	1	0	0	0	0	0	0	0	0	0	0	0	0	0	0	0	0	0	0	0
Total	117	101	84	71	62	50	41	36	31	28	24	17	16	12	10	10	8	6	5	4	4	3	3	0

#### Levins model of population dynamics and the prediction of dynamic quantity

The extinction of a local population and the establishment of a new one are the two basic processes that determine the trend of a metapopulation [20]. Therefore, the Levins model of dynamic metapopulation of *C. obcordata* can be expressed as follows:
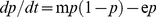
(1)where “m” and “e” are the parameters of misappropriation and extinction, respectively, and '*P* = 0 represents extinction. When the population is in a balanced state, given as *dp*/*dt* = 0, '*P* = 1–e/m (i.e., m<e), the metapopulation will ultimately trend to extinction; conversely, when e<m, the metapopulation will persistently exist [20] if the extinction rate of a local population is less than a certain marginal value. Equation (1) can be restated as follows:
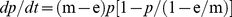
(2)


The *D* value of (m–e) in this expression can be identified as an intrinsic rate of increase [20]. The value for *C. obcordata* was −1.884, giving m<e, indicating that its population would ultimately trend to extinction.

**Figure 4:**
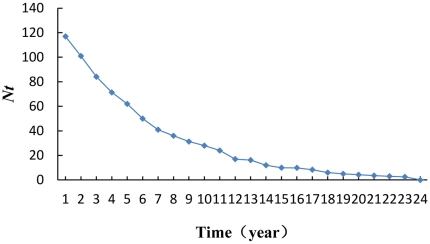
Negative growth model curve of the *C. obcordata* population.

Tables 3 and 4 indicate that *C. obcordata* is a diminishing population based on the simulation of the Leslie matrix model and the continuous descending (negative growth) model, which is consistent with data from the reproduction table and biological characteristics of the population. The quantity of each age class presented a successively descending trend — that is, the degradation trend appeared in all age classes. Further predictions based on *N_t_* = *N_t_*_–1_–*N_t_*_–1_e^−1.884^ indicate that the population size of *C. obcordata* would decrease from *N*_1_ = 117 genets per six subpopulations to *N*_23_ = 0 in approximately 23 years (Figure 4).

### Meteorological data and survival analysis of *C. obcordata*

#### Response to rainfall change

Surface flow was introduced after raining and stopped after 20 hours in the artificial sloping field. The genets under such artificial water recharge all survived as well as flowered and fruited normally, producing 47 seedlings, whereas those plants in the natural condition died during the successive episodes of drought from autumn to winter. *C. obcordata* that grew on the poolside also survived, flowered, and fruited, producing 53 seedlings. These results suggest that preserving enough water in the habitat is the key to normal *C. obcordata* growth.

#### Influence of climate change on the survival of *C. obcordata*

The average temperature from the period of 1980–2010 has increased by 1.1°C compared with the 1970s (21.68±0.24 vs. 22.69±0.24°C), and the average rainfall from the same period has decreased by 11.49 mm compared with the 1970s (1773.67 vs. 1762.18 mm) (see Table S1, data from the Guangdong Weather Archives). The change in weather data over 40 years suggests that the hydrological cycle has accelerated along with climate warming, and the severely uneven temporal and spatial distribution of water has brought about increasingly more severe drought — in particular, seasonal and local drought has become extraordinary. The temperature has gradually ascended, high temperature weather lasts for a long time, the rainfall pattern is frequently fluctuating, relative humidity is decreasing, and evaporation is advancing, further yielding successive episodes of drought in most years from autumn to winter, even extending to spring. Such serious changes in weather have resulted in significant fluctuations in the capacity of stream water in the mountains. Although temperature rise is known to increase rainfall, this study found that rainfall decreased as temperature increased, which was correlated with diminished relative humidity and increased evaporation; these factors increased the frequency of drought (“Report on Dealing with Climate Change and Strengthening Weather Disaster Prevention and Reduction Ability,” Guangdong Provincial Government). Such weather pattern caused brook blanking as well as flashfloods; in addition, it has directly changed the habitat as well as threatened the subsistence and development of *C. obcordata*. The survival curve of each age class in the life table agreed with the change in weather data, particularly with the rainfall change and especially in the pre-reproduction and reproduction phases. The survival rate was higher during the years with more rainfall and lower in those with less. The survival rate fluctuated along with rainfall. The survival rates of all age classes suggest that the population had experienced distinct weather events per age class before entering its current age class.

## Discussion

The age pyramid and population growth trend of *C. obcordata* can both be deduced using its life table. The Leslie matrix model can predict the dynamic changes in quantity and structure of a population well; it is an effective means of predicting trends, especially for a descending population such as *C. obcordata*. The continuous descending model *N_t_* = *N_t_*_–1_–*N_t_*_–1_e^−1.884^ can be used to predict the dynamic trend of a population, and its results were congruent to the findings from the spatial pattern test, age pyramid, and Leslie matrix model of this species. After calculation, m<e from the Levins model indicated that the metapopulation of *C. obcordata* would ultimately disappear from Luofu Mountain.

The dynamic quantity trend of the plant population is the product of the interaction between the survival capability of individual plants and the outer environment [21]. The age structure, life table, and survival curve of a plant population under different habitats not only reflect the actual status of the population but also exhibit the resistant relationship between the plant population and the environment [22]. Following this, the age structure, life table, and survival curve of *C. obcordata* reflect its actual status, and the predicted population size trend suggests that the fact that its reproductive seedlings and plants are unable to withstand drought and adapt to weather changes is responsible for its resistant relationship with the environment. Climate warming results in rainfall imbalance, which is an irreversible trend over the short term. As it is difficult to stabilize the growth of *C. obcordata* in conditions with unstable water currents, stabilizing this population forfeits its general conditions.

Due to the rapid development of modern industry, large amounts of CO_2_ have been released into the environment, thereby increasing surface temperature, causing the greenhouse effect, and consequently triggering global warming. Temperature rise has changed the long-term adaptability of *C. obcordata* to temperature. In response to high temperature, the plant needs to reduce its cell water capacity, increase the density of its sugar or salt content, slow down its metabolic speed, and engender vigorous transpiration to avoid overheating. However, a reduction in rainfall and extended drought caused by the significantly irregular fluctuations of rainfall both within a year and over time follow the high temperature, which prevents *C. obcordata* from withstanding drought and high temperature, especially due to the undeveloped defense mechanism of its seedlings or newborn leaves, its underdeveloped root system, and its inefficient water imbibition. Moreover, the plant consumes a considerable amount of nutrients after fruition, thereby diminishing its capacity to resist drought. Previous research has shown that the seedlings and plants of *C. obcordata* collectively died after fruiting under the dual impact of reduced water content in the environment and increased inter-water consumption [13]. This species requires a high number of seedlings for replenishment to enter the next age class successfully. The change in rainfall patterns caused by weather warming has been found to kill seedlings and reduce the viability of mature genets. As a result, seed production, seedling growth, and population replenishment prove to be difficult, leaving the population to trend toward senescence.

Vegetation and climate have a remarkable coupling relationship [23]. Climate change is the primary long-term force behind regional vegetation, which will be principally affected by temperature and rainfall. A sudden increase and change in these factors over a short period will significantly affect the species inhabiting such vegetation. An increase in temperature is known to increase water consumption, which in turn causes drought [24]; such water stress triggers the pores of *C. obcordata* to shut, causing the rates of transpiration and photosynthesis to decrease so as to avoid leaf dehydration and reduce the accumulation of dry matter [25]. The abovementioned factors could negatively influence each age-class genet of *C. obcordata* and disturb its long-term adaptability to the environment during its entire life cycle. This study has determined that *C. obcordata* is extremely sensitive to water change, and the findings have confirmed that the rainfall imbalance caused by climate warming may expedite the extinction of the species.

## Methods

### Biological characteristics

Wild populations of *C. obcordata* in Luofu Mountain (114°05′E, 22°34′N; elevation, 100–500 m) in Huizhou, Guangdong, and artificial populations in the National Orchid Conservation Center of China (Shenzhen,114°10′E, 22°35′N; elevation, 50 m) were evaluated from March 2010 to May 2011 (Figure S1). Six natural subpopulations of *C. obcordata* were observed, and the growth mode of each genet, the quantity of persistent stems, and the growth as well as the flowering and fruiting status of leafed ramets were recorded. Genet age was confirmed via the quantity of stems because each genet can only grow one new ramet annually. The flowering quantity of each age-class genet, the number of flowers on each inflorescence, and the fruiting quantity were calculated. The genet reached sexual maturity, and statistical data on the seedlings in the 1-year age class were confirmed to calculate the offspring quantity of the genet [16].

All necessary permits were obtained for our field studies. The locations for our field studies are not private lands but protected areas, controlled by the Forestry Bureau of Guangdong Province. We have obtained a valid permit from this authoritative organization (see Permit S1). The tested genets of *C. obcordata* were all artificially cultured, and none of them was collected from the field. Our field observations did not collect any plant specimen nor animal or even insect. Although *C. obcordata* is not a rare plant (no endangered plant), it is facing threatened and needs to be protected.

### Detection of reproductive mechanism

Biological pollination of *C. obcordata* was carried out in fields [16], [26] until all flowers faded or fruited, and the fruiting rate of natural pollination was calculated statistically. The mating system was investigated as described below at the National Orchid Conservation Center of China. During the flowering period of *C. obcordata*, 40 sample sites were set up and 10 flowers from each sample site were tested under the following treatments: (1) flowers were bagged before blooming until all faded or fruited and the fruiting rate was calculated; (2) flowers were bagged before blooming, pollinia were placed on the stigmata after blooming, the flowers were bagged again until all flowers faded or fruited, and the fruiting rate was calculated; or (3) flowers were bagged before blooming, pollinium was placed on the stigmata of flowers from different genets and bagged again until all flowers faded or fruited, and the fruiting rate was calculated [26].

### Dynamic analysis of population size

#### Formation of the static life table

Based on the trait of single catenulate growth with the obvious space–time mark of *C. obcordata*, the static life table was prepared using the space-to-time method [12], [27]–[29]. It includes the following: *X*, the age class; *l_x_* (survival rate), the standard survival quantity (1000) at the beginning of *X* age class; *d_x_* (death quantity), the standard death quantity from *X* age class to *X*+1 age class; *q_x_* (death rate), the death rate of the genets from *X* age class, *q_x_* = *d_x_/l_x_*×1000; *L_x_*, the average quantity of the genet from *X* age class to *X*+1 age class, *L_x_* = (*l_x_*+*l_x_*_+1_)/2; *T_x_*, the total quantity of the genet from *X* age class onward, *T_x_* = *L_x_*+*L_x_*_+1_+*L_x_*_+2_+,…; *e_x_* (life expectation), the life expectation of the genet that entered *X* age class, *e_x_* = *T_x_*/*l_x_*; *a_x_* (survival quantity), the actual survival quantity at the beginning of *X* age class (genet number/500 m^2^); and *K_x_*, the disappearance rate of the population, *K_x_* = ln*l_x_*–ln*l_x_*_+1_.

#### Formation of the survival curve and death rate curve

These curves were plotted via the individual quantity of each age class compared with time to describe the death rate at a specific age class. The death rate curve was drawn with death rate in the *y*-coordinate and age class in the *x*-coordinate, whereas the survival curve was drawn with the log value of survival quantity (log value of *l_x_*) in the *y*-coordinate and age class in the *x*-coordinate [18].

#### Formation of the population reproduction table

The population reproduction table [12] includes the following: *X*, the age class; *l_x_*, the survival rate at *X* age class; and *m_x_*, the average quantity of offspring of the genet from *X* age class (based on actual tested quantities of mature genet and 1-year seedlings). The net reproductive rate of the population, *R_o_* = Σ*l_x_m_x_*, the intrinsic rate of increase, *r_m_* = ln*R_o_/T*, the finite rate of increase, *λ* = e*^r^*, and the generation span, *T* = Σ*Xl_x_m_x_/*Σ*l_x_m_x_* [12], were also calculated.

#### Structure of the Leslie matrix model and prediction of dynamic quantity

The total survival rate *P_x_* (from *X* age class to *X*+1 age class) was calculated using the survival rate of the life table, *P_x_* = *L_x+_*_1_/*L_x_* = (*l_x_*_+1_+*l_x_*_+2_)/(*l_x_*+*l_x_*_+1_). The average quantity of offspring (*f_x_*) generated at *X* age class and that which survived at *X*+1 age class were calculated using the reproduction value, *f_x_* = *P_x_*×*m_x_*. Lastly, the quantity and age distribution of the population after unit time interval were calculated from the quantity and distribution of the population: *N_t_*_+1_ = *M*·*N_t_* = *M* ^(*t*+1)^·*N_o_*, where *M* is the Population Projection Matrix [12], [30]:
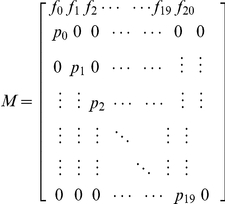



#### The Levins model of single metapopulation dynamics

Due to the spatial segregation of the population caused by its growth characteristics and existing habitat, the interaction dynamics of *C. obcordata* requires that the individuals be spread out. Based on the current fragmental situation of its habitat, the Levins model of metapopulation dynamics was utilized to make qualitative and quantitative predictions [20].

### Climate change and growth of *C. obcordata*

#### Analysis of weather data

The weather data used in this study were obtained from the Weather Archives of Guangdong Province. The time series of collected data corresponded with the largest survival age of the genet in population of *C. obcordata*, and the estimated period was from 1998 to 2010. The trend of weather change and change in weather circumstances in the distribution of *C. obcordata* were studied using weather data, including annual average temperature and rainfall. The relationship between the change in rainfall and the quantity trend of the population was analyzed in integration with the static life table.

#### Detection of influence of water-flow variation on the growth of *C. obcordata*

Artificial simulation was carried out at the National Orchid Conservation Center of China in March 2010 to test the effects of variation in water flow on *C. obcordata* growth: 40 sample sites were set up in an artificial sloping field of 85% shadow where surface flow could occur, with each sample site comprising five genets (all 4–5 years old), and 20 sample sites were set up along the poolside of a stable water storage system, with each sample likewise composed of five genets (all 4–5 years old). After normal growth under artificial management, 20 sample sites from the artificial sloping field were continuously watering; the others sites were left to grow under natural conditions. The growth statuses in different water conditions were observed and recorded.

## Supporting Information

Figure S1 **Study area in Guangdong province, China.** (**A**) Site of *C. obcordata* in Luofu Mountain. (**B**) Site of control test of *C. obcordata* in Shenzhen.
(TIF)

Permit S1 **The permit for our field studies from Forestry Bureau of Guangdong province, China.**
(TIF)

Table S1 **The data of average temperature and rainfall from 1970 to 2010.**
(DOC)
