# Supplementary figures and images for: Predicted Disappearance of Cephalantheropsis obcordata in Luofu Mountain Due to Changes in Rainfall Patterns
Source: PLoS One. 2012 Jan 10;7(1):e29718. doi: 10.1371/journal.pone.0029718 (PMC3254617; doi:10.1371/journal.pone.0029718)

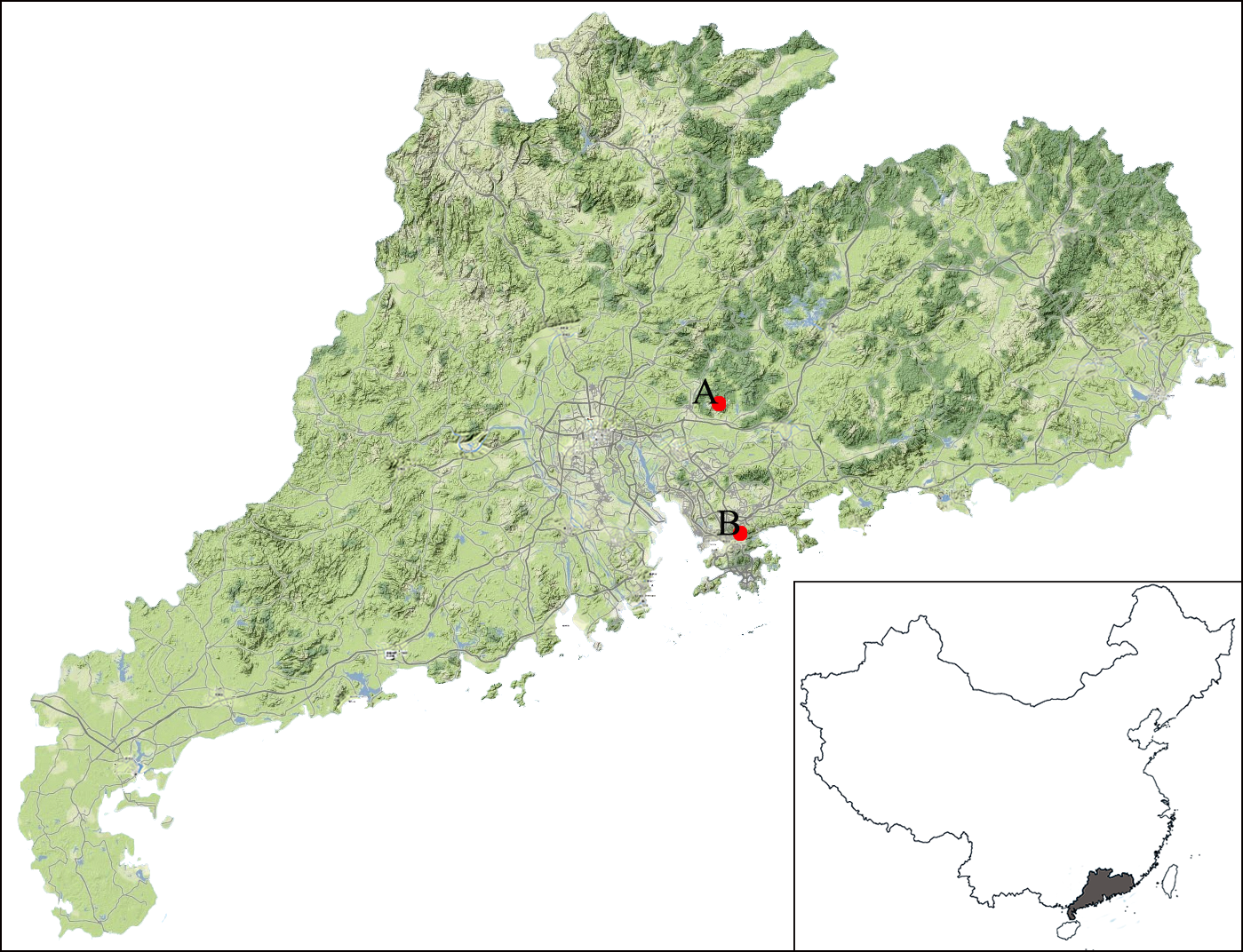

Supplement: Figure S1 — Study area in Guangdong province, China. (A) Site of C. obcordata in Luofu Mountain. (B) Site of control test of C. obcordata in Shenzhen. (TIF) [file pone.0029718.s001.tif]

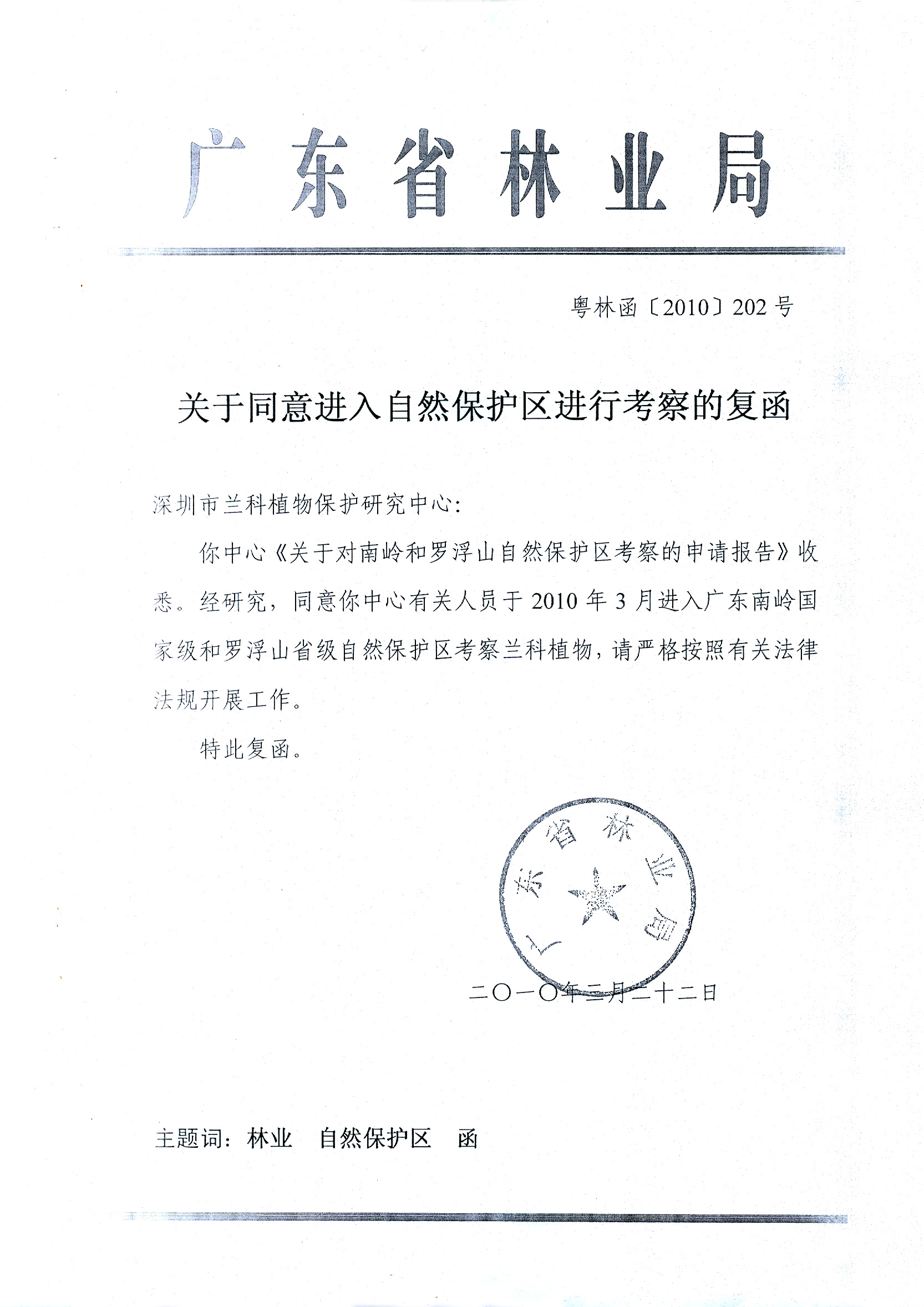

Supplement: Permit S1 — The permit for our field studies from Forestry Bureau of Guangdong province, China. (TIF) [file pone.0029718.s002.tif]
