# Supplementary material for: Predicted Disappearance of Cephalantheropsis obcordata in Luofu Mountain Due to Changes in Rainfall Patterns
Source: PLoS One. 2012 Jan 10;7(1):e29718. doi: 10.1371/journal.pone.0029718 (PMC3254617; doi:10.1371/journal.pone.0029718)
Supplement: Table S1 — The data of average temperature and rainfall from 1970 to 2010. (DOC) [file pone.0029718.s003.doc]

**Table S1.** The data of average temperature and rainfall from 1970 to 2010.

| **Year** | **Average temperature (℃)** | **Rainfall (mm)** |
| --- | --- | --- |
| 1970 | 21.5 | 1522.8 |
| 1971 | 21.5 | 1532.2 |
| 1972 | 21.7 | 1701.4 |
| 1973 | 22.0 | 2318.0 |
| 1974 | 21.6 | 1514.9 |
| 1975 | 21.6 | 2194.5 |
| 1976 | 21.3 | 1900.9 |
| 1977 | 22.1 | 1377.8 |
| 1978 | 21.7 | 1854.1 |
| 1979 | 21.8 | 1820.1 |
| 1980 | 22.1 | 1598.6 |
| 1981 | 21.9 | 1872.3 |
| 1982 | 21.8 | 1504.8 |
| 1983 | 21.6 | 2347.2 |
| 1984 | 21.3 | 1634.9 |
| 1985 | 21.4 | 1833.9 |
| 1986 | 21.8 | 1831.7 |
| 1987 | 22.5 | 1795.5 |
| 1988 | 21.5 | 1915.4 |
| 1989 | 22.0 | 1662.1 |
| 1990 | 22.2 | 1410.0 |
| 1991 | 22.6 | 1343.3 |
| 1992 | 21.7 | 1875.1 |
| 1993 | 21.9 | 1894.4 |
| 1994 | 22.4 | 1619.1 |
| 1995 | 21.7 | 1456.0 |
| 1996 | 22.1 | 1501.3 |
| 1997 | 22.0 | 2049.1 |
| 1998 | 22.9 | 1950.4 |
| 1999 | 22.6 | 1539.5 |
| 2000 | 22.5 | 2310.6 |
| 2001 | 22.7 | 2320.5 |
| 2002 | 23.0 | 1325.3 |
| 2003 | 22.7 | 1609.4 |
| 2004 | 22.6 | 1173.3 |
| 2005 | 22.4 | 1709.2 |
| 2006 | 22.9 | 2570.9 |
| 2007 | 22.9 | 1910.3 |
| 2008 | 22.3 | 1936.8 |
| 2009 | 23.0 | 1621.9 |
| 2010 | 22.6 | 1565.7 |
